# Performance of a brief geriatric evaluation compared to a comprehensive geriatric assessment for detection of geriatric syndromes in family medicine: a prospective diagnostic study

**DOI:** 10.1186/s12877-018-0761-z

**Published:** 2018-03-13

**Authors:** Yolanda K. Mueller, Stefanie Monod, Isabella Locatelli, Christophe Büla, Jacques Cornuz, Nicolas Senn

**Affiliations:** 1 University Institute of Family Medicine, Department of Ambulatory Care and Community Medicine (DACCM), University of Lausanne, Rue du Bugnon 44, 1011 Lausanne, Switzerland; 2 Public Health Office of the Canton of Vaud, Av. des casernes 2, 1014 Lausanne, Switzerland; 3 Department of Geriatrics, University of Lausanne, Ch. de Mont-Paisible 16, 1011 Lausanne, Switzerland; 4 Institute of Social and Preventive Medicine, University of Lausanne, Rue de la Corniche 10, 1010 Lausanne, Switzerland

**Keywords:** Brief geriatric evaluation, Geriatric syndrome, Diagnosis, Family medicine

## Abstract

**Background:**

Geriatric syndromes are rarely detected in family medicine. Within the AGE program (active geriatric evaluation), a brief assessment tool (BAT) designed for family physicians (FP) was developed and its diagnostic performance estimated by comparison to a comprehensive geriatric assessment.

**Methods:**

This prospective diagnostic study was conducted in four primary care sites in Switzerland. Participants were aged at least 70 years and attending a routine appointment with their physician, without previous documented geriatric assessment. Participants were assessed by their family physicians using the BAT, and by a geriatriciant who performed a comprehensive geriatric assessment within the following two-month period (reference standard). Both the BAT and the full assessment targeted eight geriatric syndromes: cognitive impairment, mood impairment, urinary incontinence, visual impairment, hearing loss, undernutrition, osteoporosis and gait and balance impairment. Diagnostic accuracy of the BAT was estimated in terms of sensitivity, specificity, and predictive values; secondary outcomes were measures of feasibility, in terms of added consultation time and comprehensiveness in applying the BAT items.

**Results:**

Prevalence of the geriatric syndromes in participants (N=85, 46 (54.1%) women, mean age 78 years (SD 6))ranged from 30.0% (malnutrition and cognitive impairment) to 71.0% (visual impairment), with a median number of 3 syndromes (IQR 2 to 4) per participant. Sensitivity of the BAT ranged from 25.0% for undernutrition (95%CI 9.8% - 46.7%) to 82.1% for hearing impairment (95%CI 66.5% - 92.5%), while specificity ranged from 45.8% for visual impairment (95%CI 25.6–67.2) to 87.7% for undernutrition (76.3% to 94.9%). Finally, most negative predictive values (NPV) were between 73.5% and 84.1%, excluding visual impairment with a NPV of 50.0%. Family physicians reported BAT use as per instructions for 76.7% of the syndromes assessed.

**Conclusions:**

Although the BAT does not replace a comprehensive geriatric assessment, it is a useful and appropriate tool for the FP to screen elderly patients for most geriatric syndromes.

**Trial registration:**

The study was registered on ClinicalTrials.gov on February 20, 2013 (NCT01816087).

**Electronic supplementary material:**

The online version of this article (10.1186/s12877-018-0761-z) contains supplementary material, which is available to authorized users.

## Background

Population ageing and increasing numbers of patients with multimorbidity are major challenges faced by health services in Western societies. In this context, the traditional disease-centered model of care is increasingly recognized for its limits when managing elderly multimorbid patients [1, 2]. A key concept in the management of elderly patients is “geriatric syndromes”, which are defined as “multifactorial health conditions that occur when the accumulated effects of impairments in multiple systems render [an older] person vulnerable to situational challenges” [3]. Geriatric syndromes may be due to multiple causes, but the main point is that they can be managed without a full understanding of the underlying pathologies [4]. Furthermore, geriatric syndromes are directly associated with functional decline [5]. Caring for elderly patients by assessing and managing geriatric syndromes, rather than only looking for a specific disease therefore corresponds much better to a patient-centered approach, as it targets the patients’ independence [6], a central determinant of their quality of life [7].

The concept of geriatric syndromes was mostly developed by geriatricians, and syndromes traditionally identified by a comprehensive geriatric assessment performed by trained health professionals [8]. However a large proportion of elderly patients does not benefit from such an assessment as their contact with the health care system is limited to their family physician (FP) [9]. Indeed, identification of geriatric syndromes is rarely performed in a systematic and standardized way by family physicians [10, 11]. The AGE program (for Active Geriatric Evaluation) was set up to develop both a screening tool for detection of geriatric syndromes and a management tool that includes management strategies for each detected syndrome, for use in family medicine. Based on a literature review, eight geriatric syndromes were identified for their particular relevance in family medicine, their association with functional decline, their prevalence, clinical significance, feasibility of screening in family medicine and availability of effective interventions [12]. These include: cognitive impairment, mood impairment, urinary incontinence, visual impairment, hearing loss, undernutrition, osteoporosis and gait and balance impairment. A brief assessment tool was constructed, based on simple validated tests to detect each of these geriatric syndromes [12]. As detailed in our conceptual framework [12], screened syndrome should then be confirmed by additional investigations and a management plan be developed, as part of a global evaluation of the patient, that also includes the assessment of functional status, comorbidities and patient preferences within his broader social and spiritual context.

Increasingly, tools for rapid geriatric assessment in primary care are being developed and tested [13, 14], mostly with the objective of identifying frail or vulnerable individuals. By contrast, the aim of the active geriatric evaluation evaluated here is not only to identify patients requiring referral to more specialized geriatric care, but also to promote first-line management by FPs themselves. Most available tools target similar geriatric syndromes [15–17], although we decided not to include fatigue, frailty and sarcopenia as such in our conceptual framework. While we acknowledge that these are also important concepts in the management of elderly persons, their true meaning remains difficult to grasp for FPs, in the lack of a common definition and/or direct operational consequences for the patient.

In the present study, the AGE program aimed to estimate the diagnostic performance of this brief assessment tool compared to a comprehensive clinical geriatric assessment.

## Methods

This prospective diagnostic study compared the ability to detect eight chosen geriatric syndromes by FPs using the brief assessment tool (BAT) and by geriatricians using a comprehensive assessment. Patients were eligible if aged 70 years or older, routinely followed at one of the four recruitment sites, they have a good understanding of French or can come to the consultation with a translator and able to provide informed consent. Patients who had already benefited from a previous geriatric assessment were excluded.

The study was conducted at four sites: (1) the primary care outpatient clinic of the University of Lausanne (Department of ambulatory care and community medicine), (2) a private outpatient clinic in Lausanne and (3, 4) two private practices in two villages of the Canton of Vaud, Switzerland. Participating FPs were either family medicine residents, under the supervision of senior registrars, or specialists in general internal medicine. In Switzerland, geriatricians are specialists in general internal medicine, with an additional geriatric subspecialty corresponding to 3 years specific training. Geriatricians may be active in acute hospital, rehabilitation, long term, as well as ambulatory care settings. Geriatricians involved in the study provided outpatient consultations to patients usually referred by their FP. Potentially eligible patients were identified by the care site administrative staff before a planned consultation. On the day of the consultation, a study staff-member checked inclusion criteria, provided information on the study, collected informed consent and made a specific appointment with the geriatrician at the family practice within the following two months. The FP then conducted the routine consultation using the BAT. Patients who missed their appointment with the geriatrician received a written reminder to contact the study staff. Geriatricians were unaware of the results of the FP’s BAT-based assessment when performing their own assessment. FPs subsequently received a written report of the comprehensive geriatric assessment.

The following eight geriatric syndromes were chosen for detection: cognitive impairment, mood impairment, urinary incontinence, visual impairment, hearing loss, undernutrition, osteoporosis and gait and balance impairment. In addition, functional ability was assessed. Details on the BAT are published elsewhere [12]. Tests to assess the syndromes by the BAT and comprehensive geriatric assessment, respectively, are detailed in Table 1. Use of the BAT was considered complete if the FP completed the specified items for each syndrome.

**Table 1:** Items of the brief assessment tool and the comprehensive geriatric evaluation, respectively, by geriatric syndrome

	Brief assessment tool by the family physician	Comprehensive Geriatric assessment by geriatrician
General		Social context
Functional ability	4 questions about ADL	ADL and IADL
Cognitive impairment	MiniCog (3 words recall and clock test)	History – heterohistory MMSE, clock test, delirium assessment, ev. additional neuropsychological examinations
Mood impairment	2 questions	GDS
Urinary incontinence	4 questions	Full history, bladder-scan
Gait and balance	Observation/falls during past year	History, falls during past year, Tinetti's POMA, clinical examination, risk factors, orthostatic hypotension
Visual impairment	Reading the newspaper	Snellen scale, visual field
Hearing impairment	Whispering test	History, whispering at 30 / 60 cm
Undernutrition	Weight loss in past 1 and 6 months	History, weight loss in past 1 and 6 months, MNA score, BMI
Osteoporosis	Height loss, wall-occiput, rib-pelvis	Height loss, wall-occiput, rib-pelvis

Abbreviations: *ADL*, Activities of Daily Living [25], *BMI* Body-mass-index, *FP* Family physician, *GDS* Geriatric Depression Scale [26], *IADL* Instrumental Activities of Daily Living [27], *MMSE* Mini-Mental State Examination [28], *MNA* Mini-Nutritional Assessment, *POMA* Performance-Oriented Mobility Assessment [29]

The comprehensive geriatric assessment was performed by geriatricians and considered as the reference test. Comprehensive geriatric assessment is a structured evaluation that aims a) to identify health-conditions relevant to elderly patients; b) to determine the functional and social impact of these conditions; c) to evaluate the patients’ resources, needs and preferences; d) to propose an adapted care plan based on these identified needs and preferences. The identification of geriatric syndromes through this assessment is based on validated clinical tests, without systematic use of confirmatory investigations such as MRI or laboratory tests. This broad approach has been shown to reduce morbidity, mortality and the need for institutionalization [18, 19]. While the validity of the screening tests used in the comprehensive geriatric assessment has been established [12], aspects of test reliability have rarely been explored. Therefore, agreement and reliability between geriatricians were previously investigated by the AGE program [20]. Reliability was good to excellent for functional ability, cognitive impairment, hearing impairment, osteoporosis, incontinence (three-way intraclass correlation: 0.6 ≤ 3WICC < 0.8) and mood impairment (3WICC ≥ 0.8). Reliability was moderate for risk of fall and imbalance (0.4 ≤ 3WICC < 0.6), and poor for visual impairment and malnutrition (3WICC < 0.2). These characteristics were judged sufficient to use it as the reference consultation for detection of geriatric syndromes, except for visual impairment and malnutrition which should be assessed in a setting with access to longitudinal medical records (for objective weight loss assessment for example) [20].

When recording evaluations for each syndrome, the FP’s and geriatricians could choose one of three categories, for example absent/possible/present or absent/moderate/severe. Results of each syndrome evaluation were then dichotomized into absent/suspected syndrome, as detailed elsewhere [20]. Patients with intermediate results usually require additional investigations and for this study were considered as a positive result. If evaluation of a specific syndrome was missing in either BAT or geriatric assessment, the observation were excluded (complete records analysis). Data was collected on standardized paper questionnaires by the FP and geriatrician, single-entered into EpiData v3.1 and analyzed by Stata IC 14.1 (College Station, USA). Basic, instrumental and total activities of daily living, as well as the number of detected geriatric syndromes were described by median, interquartile range, and box plots.

Initial sample size was calculated to estimate an expected sensitivity of 90% with a lower bound of the 95% confidence interval (95CI) being larger than 65% with a 95% probability. This corresponded to 31 individuals with the condition (based on the comprehensive geriatric assessment) and 124 without the condition, if using an estimated prevalence of 20% [21]. Because of slow recruitment, the final sample size was reduced to at least 24 patients with the condition, which was judged to give acceptable precision (lower bound of the 95CI decreased from 65% to 60%).

## Results

**Fig. 1:**
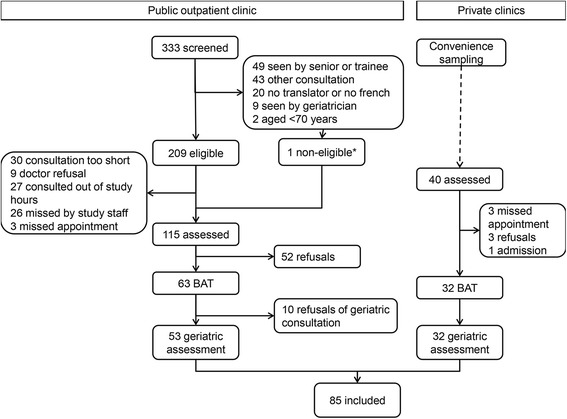
Patient flow, AGE2 study. BAT = Brief assessment tool; *: One patient assessed under 70 years, who had reached 70 at the time of the geriatric assessment

Of the 85 patients included between March 2013 and December 2014, 32 (37.7%) were included in private practices and 53 (62.4%) at the University outpatient clinic. The detailed patient flow is available for the latter, whereas in the private practices, patients were selected by convenience by the FP (Fig. 1). The main reasons for eligible patients not being included were patient refusals (65, including 55 initial refusals for the entire study and 10 drop-outs who refused the geriatric assessment), not being assessed by the study staff (56), and physicians not able to perform the BAT (39, including 30 because of lack of consultation time to include the BAT*).* Demographic characteristics were representative of the elderly population in the canton of Vaud [22], and functional status and self-rated health of included patients were comparable with that of community-dwelling Swiss elderly population [23].Patient characteristics are shown in Table 2. There were slightly more females than males included. Mean age was 78 years (SD 6). The 33 patients not born in Switzerland had been living in Switzerland for a mean of 43 years (SD 16 years). Most patients considered themselves in good or very good health, although more than half of them were considered vulnerable by the geriatrician (Table 2). Proportion of vulnerable or dependent patients was similar between private practices and outpatient clinic (chi^2^*p* = 0.485), although there were more females (68.8% vs. 45.3%; chi^2^*p* = 0.035) and mean age was higher (80 years (SD 7) in private practices vs. 76 years (SD 5) in outpatient clinic; t-test *p* = 0.011).

**Table 2:** Patient characteristics, AGE2 study (*N* = 85)

	Number	Percent
Gender		
- Female	46	54.1
- Male	39	45.9
Age category (in years)		
- 69 to 74	34	40.0
- 75 to 84	37	43.5
- 85 to 94	14	16.5
Country/region of birth		
- Switzerland	52	61.2
- European region except Switzerland	19	22.4
- Outside European region	14	16.5
Achieved education level (8 missing)		
- Primary school (9 years)	21	27.3
- Secondary school (12 years)	29	37.7
- Superior education (secondary school + at least 3 years)	27	35.1
BMI category (7 missing)		
- Underweight (< 18)	2	2.6
- Normal (18–25)	28	35.9
- Overweight (25–30)	25	32.1
- Obese (> 30)	23	29.5
Cardiovascular risk factors		
- Hypertension (3 missing)	57	69.5
- Hypercholesterolemia (4 missing)	47	58.0
- Diabetes (3 missing)	21	25.6
Cardiovascular disease (4 missing)	25	30.9
Respiratory disease (4 missing)	17	21.0
Cancer (3 missing)	7	8.5
Number of different medications (2 missing)		9.4
- 0 to 5	47	56.6
- 6 to 10	24	28.9
- 11 to 15	10	12.1
- > 15	2	2.4
Wearing glasses (4 missing)	69	85.2
Wearing hearing aid (4 missing)	17	21.0
Self-rated health (7 missing)		
- Very good	14	18.0
- Good	42	53.9
- Fair	20	25.6
- Poor	2	2.6
Global evaluation (2 missing)		
- Robust patient	32	38.6
- Vulnerable	45	54.2
- Dependent	6	7.2

The 85 BAT assessments were performed by 46 different FPs, while four geriatricians performed the comprehensive geriatric assessments, a median of 22 days after the FP appointment (IQR 9–44 days). Thirteen patients were assessed by the geriatrician more than two months after the FP appointment, but none had encountered a significant health or social problem within these two months that could have significantly affected their overall health status.

Diagnostic performance of the brief assessment tool for detecting each of the eight geriatric syndromes was estimated using positive detection by the geriatrician’s comprehensive geriatric assessment as a reference standard (Table 3; Additional file 1). Sensitivity ranged from 25.0% for undernutrition (95% CI 9.8% - 46.7%) to 82.1% for hearing impairment (95% CI 66.5% - 92.5%), while specificity ranged from 45.8% for visual impairment (95% CI 25.6–67.2) to 87.7% for undernutrition (76.3% to 94.9%). Finally, most negative predictive values (NPV) were between 73.5% and 84.1%, excluding visual impairment with a NPV of 50.0%. Negative likelihood ratios ranged between 0.2 and 0.5.

**Table 3:** Prevalence of geriatric syndromes and performance of the brief assessment tool compared to geriatricians evaluation

Syndrome	Prevalence (%)	Sensitivity (95% CI)	Specificity (95% CI)	PPV (95% CI)	NPV (95% CI)	LR+ (95% CI)	LR- (95% CI)
Functional loss	14.0	91.7 (61.5–99.8)	95.8 (88.1–99.1)	78.6 (49.2–95.3)	98.6 (92.2–100.0)	21.7 (7.1–66.5)	0.1 (0.0–0.6)
Cognitive impairment	29.8	64.0 (42.5–82.0)	67.2 (53.7–79.0)	45.7 (28.8–63.4)	81.3 (67.4–91.1)	2.0 (1.2–3.1)	0.5 (0.3–0.9)
Mood impairment	37.7	65.6 (46.8–81.4)	64.2 (49.8–76.9)	52.5 (36.1–68.5)	75.6 (60.5–87.1)	1.8 (1.2–2.8)	0.5 (0.3–0.9)
Urinary incontinence	43.5	76.5 (58.8–89.3)	85.4 (72.2–93.9)	78.8 (61.1–91.0)	83.7 (70.3–92.7)	5.2 (2.6–10.7)	0.3 (0.1–0.5)
Gait and balance	34.9	67.9 (47.6–84.1)	73.6 (59.7–84.7)	57.6 (39.2–74.5)	81.3 (67.4–91.1)	2.6 (1.5–4.3)	0.4 (0.2–0.8)
Visual impairment	71.1	81.4 (69.1–90.3)	45.8 (25.6–67.2)	78.7 (66.3–88.1)	50.0 (28.2–71.8)	1.5 (1.0–2.2)	0.4 (0.2–0.8)
Hearing impairment	47.6	82.1 (66.5–92.5)	86.0 (72.1–94.7)	84.2 (68.7–94.0)	84.1 (69.9–93.4)	5.9 (2.8–12.5)	0.2 (0.1–0.4)
Undernutrition	28.9	25.0 (9.8–46.7)	87.7 (76.3–94.9)	46.2 (19.1–74.9)	73.5 (61.4–83.5)	2.0 (0.8–5.4)	0.9 (0.7–7.8)
Osteoporosis	47.5	77.8 (60.8–89.9)	65.9 (49.4–79.9)	66.7 (50.5–80.4)	77.1 (59.9–89.6)	2.3 (1.4–3.6)	0.3 (0.2–0.6)

Brief assessment tool for detection of functional loss and geriatric syndromes was used by family practitioners and compared to geriatricians’ evaluation

*PPV* positive predictive value, *NPV* negative predictive value, *LR+* positive likelihood ratio, *LR-* negative likelihood ratio

According to the comprehensive geriatric assessment, almost all patients (91.2%) presented at least one geriatric syndrome, with a median number of three suspected syndromes per patient (IQR 2 to 4). Prevalence by type of syndrome ranged from 29.8% to 71.1%.The most prevalent geriatric syndrome was vision impairment, followed by hearing loss and osteoporosis. Overall, functional abilities were preserved, with a median of 13 activities of daily living (ADL) performed independently by participants out of a maximum 14 (IQR 12 to 14). Performance of screening for functional disability by four questions only was excellent compared to the detailed 14 items ADL assessment (sensitivity 91.7%, 95%CI 61.5% - 99.8%; specificity 95.8%, 95%CI 88.1% - 99.1%).

Family physicians reported BAT use as per instructions for 76.7% of the syndromes assessed. By syndrome, completeness ranged from 68.3% to 88.0%. The main reasons FPs gave for not completing the assessment were lack of time, that they forgot, or that they judged the assessment unnecessary, either because of the good general condition of the patient or because the condition was already known. When analyzing diagnostic performance restricted to items completed by the FPs there was less than 10% variation in the estimated negative predictive value (NPV) compared to the entire dataset, and none of the differences were statistically significant. In terms of feasibility, it took 20 min on average (IQR 15 to 30 min; 4 missing) to perform the BAT. Most FPs (95.2% = 79/83, 2 missing) considered the BAT adapted to their needs.

## Discussion

The BAT’s performance for detecting geriatric syndromes compared to a comprehensive geriatric assessment was satisfactory for most syndromes. Sensitivity was within the prespecified range (95%CI > 60%) for visual impairment, hearing impairment, and osteoporosis. Sensitivity estimates were from 40% to 90% for urinary incontinence, cognitive impairment, mood impairment and gait and balance impairments. Still, the negative predictive values were sufficient to reasonably exclude the considered syndromes. Specificities were above 50% (with the exception of visual impairment), which can be considered a good result, considering these were clinical tests [24], and meant to be used as screening tests that can allow some false positive results as they may be combined with more specific confirmatory tests. Screening with the BAT was feasible in routine FP consultations. Among eligible patients, only a minority were not included because of physician refusal. The time added to the consultation can be a barrier if not anticipated, but it was manageable for most FPs. They also considered the tool adapted to their needs.

This study is unique in directly comparing performance of a detailed geriatric assessment with a brief assessment by FP. Patients were directly recruited in primary care, the setting for which the BAT has been developed. While the mode of recruitment may have selected frequent users of outpatient care, thereby favoring more vulnerable patients compared to the general population, and although patients included at the University hospital outpatient clinic may not be truly representative of the general family practice, demographic characteristics, functional status and self-rated health of included patients were comparable with that of community-dwelling elderly population of Switzerland.

The main limitations of this study reside in the limited sample size, the imperfectness of the reference standard, and the non-simultaneous assessments by FP and geriatricians. First, our sample size was limited, only allowing us to make a broad estimate of diagnostic performance. In addition, the hypothesis of a 90% sensitivity was too optimistic. Second, the BAT was compared with a comprehensive geriatric assessment as reference standard, which cannot be considered a perfect gold standard. Indeed, geriatricians’ and FPs’ perspectives may somewhat differ within the context of a geriatric assessment [14]. Therefore, some misclassification is likely, altering the estimated diagnostic performance of the BAT, especially for items with low intraclass correlation such as undernutrition and visual impairment [20]. For example, the many patients classified as at risk of undernutrition by the geriatrician that were not considered undernurished by their FP were counted as “false-negatives”, causing an underestimation of the sensitivity of the FP’s assessment. Similarly, patients wearing glasses were considered to have light vision impairment, even if their actual visual performance was satisfactory, leading to a low number of patients without visual impairment and therefore an imprecise specificity estimate for visual impairment. Also, the comprehensive geriatric assessment was a one-shot encounter between the geriatrician and an unknown patient, compared to a longitudinal follow-up in the context of family medicine, which may actually have better reliability than the reference for some key measurements such as weight variations over time. Finally, assessments by FP’s and geriatricians were not simultaneous and the condition of the patient may have changed in-between. However, time interval between both consultations was limited, and patients who experienced a major life event between the two visits were excluded, limiting the risk of important changes of health status. In addition, previous analyses of the comprehensive geriatric assessment showed a negligible “visit effect”, corresponding to the proportion of the variance that varies from visit to visit in a single patient, except for mood disorders, where time change explained 4% of the total disagreement [20].

While previous studies often focused on one or a few specific syndromes, often requiring long assessments, the BAT targets eight of them integrated into a single tool, which is more adapted to family practice and, more importantly, to the reality of elderly patients who usually suffer from more than one condition, as also seen here. Indeed, screening for these eight syndromes might encompass most geriatric issues that are directly relevant for the FP when managing their elderly patients. While other tools are now being developed for primary care [14, 15, 17], data on validation and feasibility are still limited. In particular, the clinical utility of this approach, namely whether acting on these geriatric syndromes in the context of family medicine will slow down the functional decline of the patients, still needs to be proven. This next step will be evaluated in a clinical trial comparing the complete active geriatric evaluation, which combines the brief assessment tool with recommendations for further investigations and management options, with usual care by FPs, currently ongoing (ClinicalTrials.gov Identifier: NCT02618291).

## Conclusions

Although the BAT does not replace a comprehensive geriatric assessment, it is a useful tool appropriate for the FP. Acknowledging the limitations of both the BAT and the CGA, assessments for visual impairment and undernutrition should be further optimized for the family medicine context. Results of the BAT, considered as other clinical test results as part of a global patient evaluation, can be used to screen for patients who would benefit from additional investigations or a second more in depth assessment by a specialist.

## Additional file

Additional file 1: Cross-tabulation of brief assessment tool results, by result of comprehensive geriatric assessment. (PDF 29 kb)

## Abbreviations

ADL: Activities of daily living
AGE: Active geriatric evaluation
BAT: Brief assessment tool
CI: Confidence interval
FP: Family physician
IQR: Interquartile range
NPV: Negative predictive value
SD: Standard deviation
