# Supplementary material for: Performance of a brief geriatric evaluation compared to a comprehensive geriatric assessment for detection of geriatric syndromes in family medicine: a prospective diagnostic study
Source: BMC Geriatr. 2018 Mar 13;18:72. doi: 10.1186/s12877-018-0761-z (PMC5850979; doi:10.1186/s12877-018-0761-z)
Supplement: Supplementary file 1 — Cross-tabulation of brief assessment tool results, by result of comprehensive geriatric assessment. (PDF 29 kb) [file 12877_2018_761_MOESM1_ESM.pdf]

Additional file 1

Cross-tabulation of brief assessment tool results, by result of comprehensive geriatric assessment

Family practitioner	Geriatrician		
Functional dependency (N=83)	Little	Moderate	Important
- Little	68	1	0
- Moderate	3	7	2
- Important	0	1	1
Cognitive disorder (N=83)	None	Possible	Certain
- None	39	8	1
- Possible	18	9	1
- Certain	1	1	5
Clock test (N=78)	Normal	Limit	Pathologic
- Normal	58	3	4
- Limit	4	0	0
- Pathologic	4	1	4
Mood disorder (N=85)	None	Possible	Certain
- None	34	7	4
- Possible	14	5	6
- Certain	5	6	4
Walking disorder (N=84)	None	Light	Severe
- None	37	5	0
- Light	19	11	2
- Severe	1	5	4
Falls during past year (N=81)	No	Yes	
- No	48	8	
- Yes	8	17	
Risk of falls (N=81)	Low	Moderate	High
- Low	39	8	1
- Moderate	11	9	4
- High	3	4	2
Osteoporosis (N=77)	No	Yes	
- No	27	8	
- Yes	14	28	
Undernutrition (N=81)	Absent	At risk	Present
- Absent	50	18	0
- At risk	5	3	1
- Present	2	0	2

Visual impairment (N=83)	None	Light	Severe
- None	11	11	0
- Light	13	38	5
- Severe	0	2	3
**Hearing impairment (N=82)**	**None**	**Light**	**Severe**
- None	37	6	1
- Light	6	17	3
- Severe	0	7	5
**Urinary incontinence, any type (N=82)**	**Absent**	**Present**	
- Absent	41	8	
- Present	7	26	
